# Unveiling the Success of Awake Insertion of Supraglottic Airway Device for Ventilation in the Bronchoscopic Management of Tracheal Stenosis

**DOI:** 10.7759/cureus.54703

**Published:** 2024-02-22

**Authors:** Vipul Sharma, Harika Atluri

**Affiliations:** 1 Anesthesiology, Dr. D. Y. Patil Medical College, Hospital and Research Center, Dr. D. Y. Patil Vidyapeeth, Pune, IND

**Keywords:** laser therapy, mitomycin c (mmc), flexible fiberoptic bronchoscopy (ffb), supraglottic airway device (sad), tracheal stenosis

## Abstract

Tracheal and subglottic stenoses are inflammatory conditions that can arise from a variety of potential etiologies, most commonly as a result of iatrogenic airway injury due to endotracheal intubation. Significant stenosis requires management by endoscopy or surgical resection. We describe a case of recurrent subglottic cuff stenosis with an episode of sudden desaturation in a 25-year-old female. The management involved balloon dilatation, laser ablation, and topical mitomycin C application through a flexible fiberoptic bronchoscope. Ensuring safe gas exchange during bronchoscopy was a priority, and this was achieved by maintaining ventilation with a supraglottic airway device, which was inserted in an awake patient after adequate tropicalization of the oral cavity. The intervention successfully helped in the management of stenosis and also addressed the complication of sudden complete airway collapse due to sedation.

## Introduction

Airway stenosis is a decrease in diameter/physical narrowing of the larynx or trachea [1]. The disease has a chronic course that affects the quality of life and the severity of symptoms. The main causes of adult tracheal stenosis include inflammatory lesions such as tumors, trauma, infection, and post-intubation cuff stenosis. Even though the prevalence of post-intubation stenosis has declined since low-pressure, high-volume cuffs were introduced, still it is the most common etiology, accounting for around 75% of cases of stenosis [2]. Post-intubation stenosis has been linked to several variables, such as the use of large-size tubes, overinflation of the cuff, bucking or traction of the circuit in an intubated patient causing tube movement, and prolonged intubation. Other contributory factors may include transient or sustained hypotension, steroid use, diabetes, and any infections [3].

Though the symptoms are nonspecific, the initial symptoms to appear are cough and dyspnea on exertion (DOE). When there is a 50% reduction in tracheal diameter, the patient presents with DOE, which is the most common presentation. This can progress to dyspnea at rest when there is 75% reduction in tracheal diameter [4]. Any infection in the upper respiratory tract may cause acute exacerbation.

The pathology presents a management challenge for the doctors, particularly in deciding which kind of initial care to provide the patient with: endoscopic or open. Conventionally, open techniques including laryngotracheoplasty, cricotracheal resection, tracheotomy, and tracheal resection were the only options. In 1970, the advent of laser using carbon dioxide (CO_2_) for medical purposes led to the popularization of endoscopic management [2,5]. Recent developments in technology and instruments have increased the success rates of endoscopic procedures while lowering morbidity [6].

For patients suffering from significant lower tracheal stenosis, surgical resection can be life-saving; however, maintaining an efficient gas exchange is essential during this procedure. Even in cases when the trachea is completely blocked, gas exchange can still be accomplished with the help of extracorporeal circulation by femoral vein and artery cannulation. When a patient has severe or total tracheal stenosis, we have to thoroughly analyze the grade and location of obstruction before inducing anesthesia. Additionally, setting up a cardiopulmonary bypass should be taken into consideration to minimize the dangers associated with traditional anesthesia [7].

The airway is shared simultaneously by both the physician for the dilatation and the anesthetist, who also should maintain adequate ventilation during the bronchoscopic procedures. The upper airway is commonly accessed with a suspension microlaryngoscopy (SML). In the management of subglottic stenosis, several ventilator strategies are employed.

One of the most often used methods is intubating with a small-sized tube, which involves the surgeon operating around this endotracheal (ET) tube. Two other types of ventilation are apneic ventilation and jet ventilation. During the jet ventilation process, the surgeon can continue working as long as egress is available. Oxygenation is taken care of by the jet of air, whereas CO_2_ is washed out during passive egress [8-10].

Transnasal humidified rapid-insufflation ventilatory exchange (THRIVE) is a new advent in airway surgery. THRIVE has the advantage of extending the apnea time and increasing this method's effectiveness [11].

An alternate method to the SML approach is flexible bronchoscopy using a laryngeal mask airway (LMA) device. This technique allows the use of a CO_2_ laser that is passed through the LMA till the stenotic site, and then balloon dilatation is performed using controlled radial expansion dilators to achieve the desired results [12].

This case report describes a multimodal approach in tackling a subglottic stenosis patient by using the I-gel to form a closed system for ventilation and also giving good surgical access, which includes laser-guided incisions of the stenotic site, followed by serial balloon dilatations and the topical application of mitomycin C as an adjunctive treatment.

A naturally occurring antibiotic called mitomycin C was discovered from *Streptomyces casepitosis*. Its conventional mechanism of action as an anti-neoplastic drug involves blocking DNA synthesis, inhibiting RNA and protein synthesis, and acting as a cross-linker on DNA. It is not clear how the action works. Based on research, even a 5-minute exposure to low doses of mitomycin C can suppress fibroblast proliferation [13].

## Case presentation

A 25-year-old female (height 147 cm; weight 37 kg) presented with cough, breathlessness (Modified Medical Research Council [MMRC] grade III), and wheezing for 20 days, which were aggravated in the last five days.

The patient had a history of organophosphorus poisoning 45 days ago when she was rushed to the nearest hospital in emergency where she was intubated and a gastric lavage was performed. After stabilization, the patient was extubated after three days and subsequently discharged from the hospital.

On this admission, all the routine blood investigations conducted were normal, and the pulmonary function test showed a fixed obstructive pattern on the flow volume curve. The patient had an episode of fall in SpO_2_ in the night up to 50% on room air with tachypnea and tachycardia. The arterial blood gas analysis showed type II respiratory failure (with hypercarbia). To stabilize the patient, emergency tracheostomy was performed under local anesthesia with a 7-mm tracheostomy tube. Her saturation improved to 98% on 6 L of oxygen. Diagnostic bronchoscopy was performed on the next day, which showed subglottic tracheal narrowing, and the patient was taken up for the procedure the next day. The patient was premedicated with IV glycopyrrolate (0.004 mg/kg), midazolam (0.02 mg/kg), and injection fentanyl (2 mcg/kg), and was induced with injection propofol (2 mg/kg) and injection atracurium (0.5 mg/kg). The patient was put on volume control mode of ventilation via the tracheostomy tube, and the rigid bronchoscope was passed through the oral cavity. Tracheal stenosis was observed in the subglottic region, where balloon dilatation was performed while the patient was being ventilated via the tracheostomy. Then the patient was intubated with an 8-size ET tube, and the tracheostomy tube was removed. With a rigid bronchoscope, serial balloon dilatations were performed. The patient was shifted to the ICU for further observation. Two days later, once the patient was hemodynamically stable and had good respiratory efforts, a bronchoscopy was performed, which showed a completely open tracheal lumen with no bleeding or narrowing. She was extubated and shifted to the ward and then discharged two days later.

The patient returned to the hospital 15 days later with complaints of breathlessness and cough where she was unable to expectorate. She had a heart rate of 110 bpm, blood pressure of 110/60, and room air SpO_2_ of 97%, and inspiratory stridor was present.

**Figure 1:**
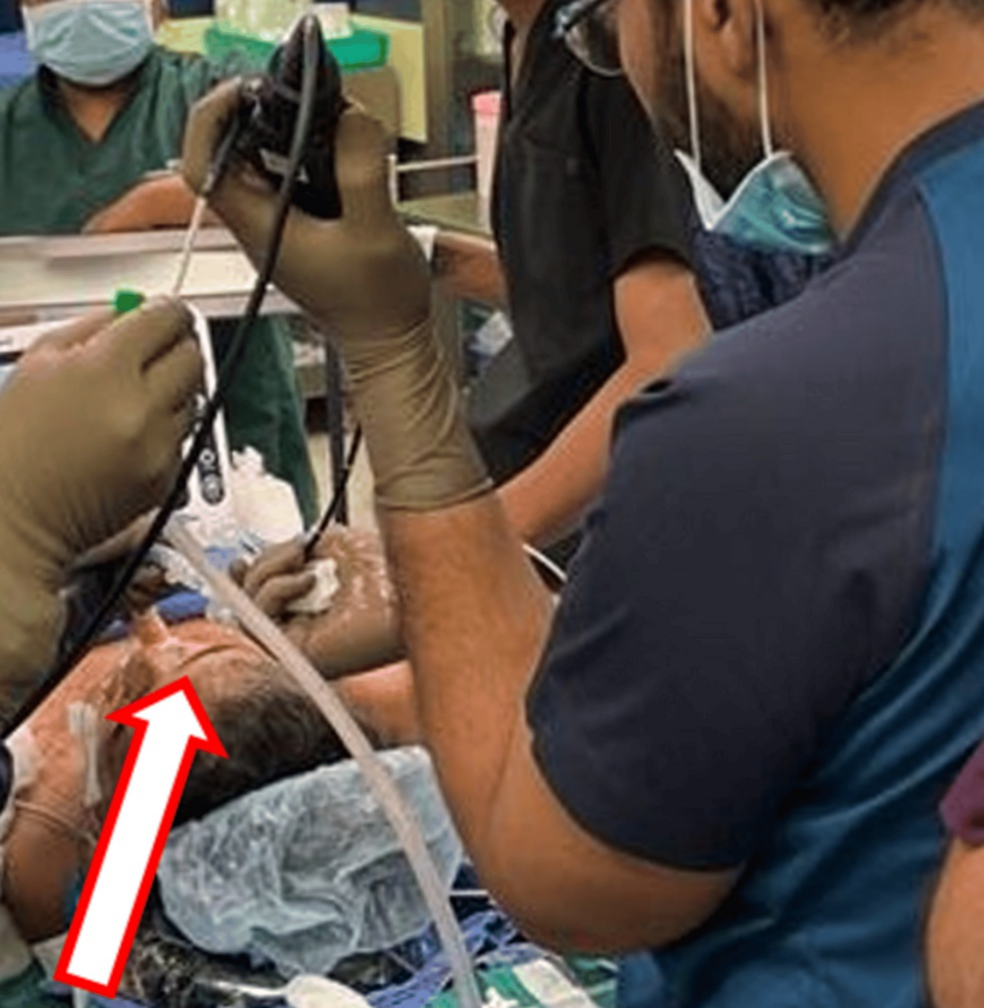
Flexible bronchoscope used in the management of a tracheal stenosis patient. This figure shows the use of flexible bronchoscope in a female patient for tracheal dilatation.

**Figure 2:**
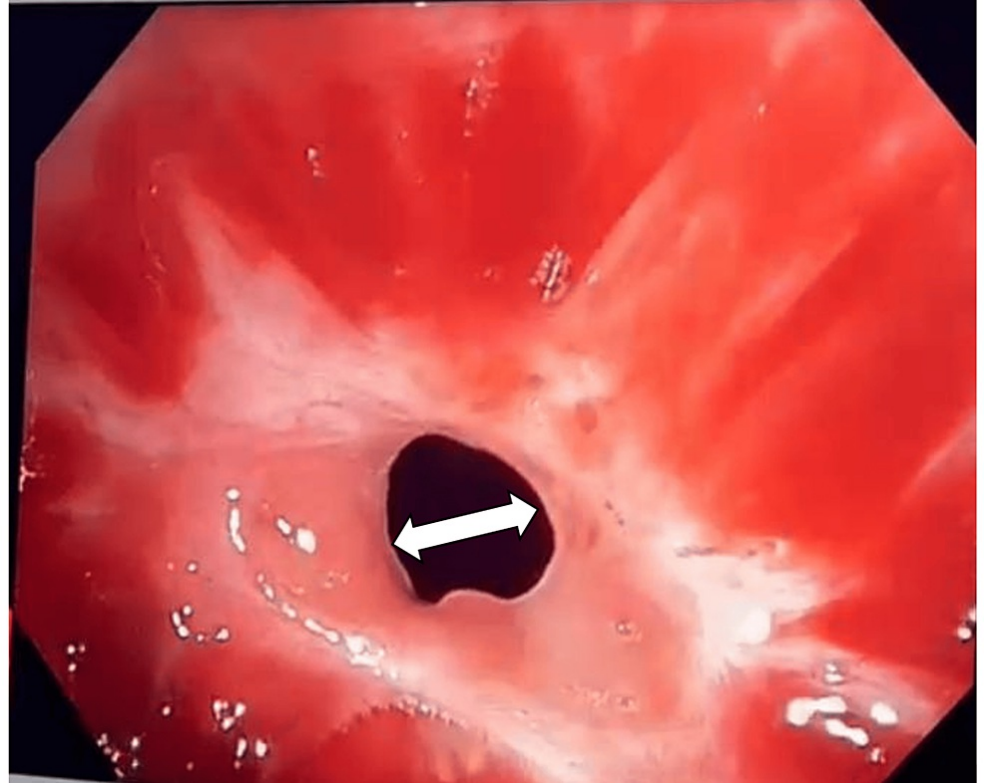
Bronchoscopic image of subglottic narrowing of the trachea at the stenotic site. This image shows the bronchoscopic view of the stenosed subglottic area of the trachea.

**Figure 3:**
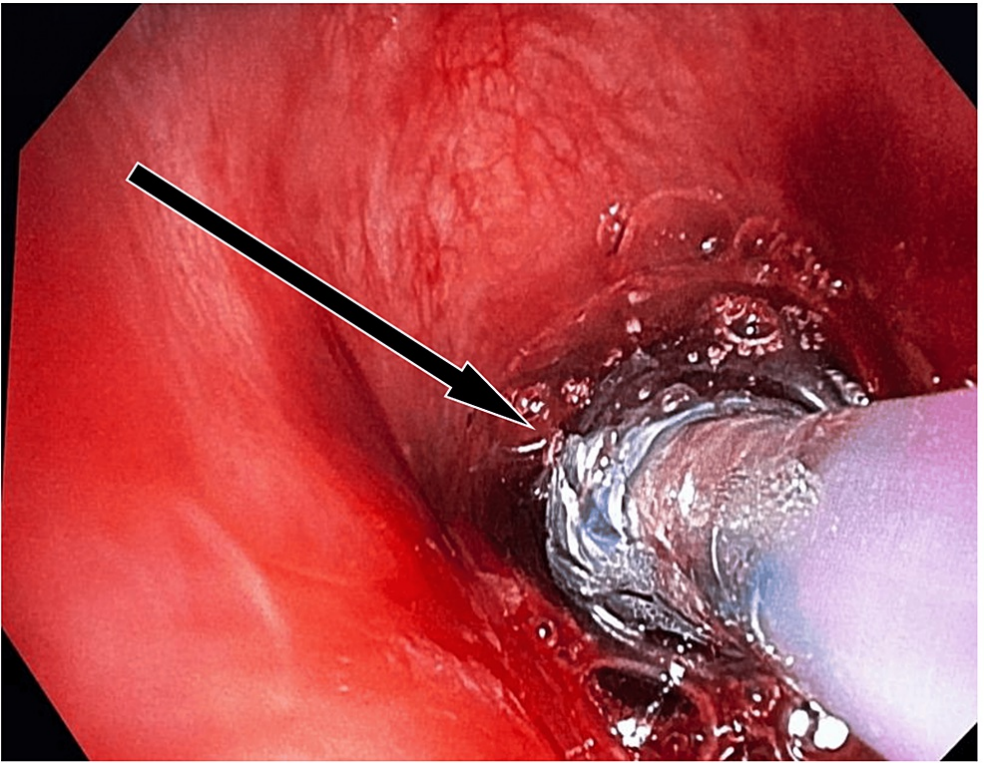
Pulmonary balloon dilatation catheter at the stenotic site. Pulmonary balloon dilatation catheter was introduced through the flexible bronchoscope, and serial dilations were performed by inflating the balloon at variable pressures.

In the operating room, the patient was re-assessed, all the routine monitors were attached, and a patent IV access was obtained. The patient was nebulized with 4 mL of 4% lidocaine. In the supine position, the patient's oropharynx was anesthetized with 10% lidocaine spray. We asked the patient to open the mouth and protrude the tongue, which was retracted medially, and intra-oral glossopharyngeal nerve block was given with 2 mL of 2% lidocaine on either side and transtracheal block was given with 4 mL of 2% lidocaine. The patient was pre-medicated with IV midazolam (0.02 mg/kg), pre-oxygenated with 100% oxygen, and injection fentanyl (2 mcg/kg) was given. After 3 minutes of pre-oxygenation awake insertion of 3 size I-gel over the supraglottis was performed, adequate ventilation without significant leak was achieved, and the patient was put on CPAP with fraction of inspired oxygen (FiO_2_) of 100%. The larynx and trachea were then visualized by passing a flexible video bronchoscope through the I-gel (Figure 1). Before the bronchoscope was passed via the glottic airway, 10 mL of 4% lidocaine was sprayed over the vocal folds via the working port to prevent laryngospasm. Severe subglottic stenosis was identified (Figure 2). A pulmonary balloon dilatation catheter was introduced. Once the catheter has reached the tracheal stenosis area even if there is a trachea collapse at the stenotic site due to sedation, it can immediately be dilated by inflating the balloon and the airway be protected. Then the patient was induced with propofol (2 mg/kg) and atracurium (0.5 mg/kg). The patient was put on volume control mode with FiO_2_ of 100%, and propofol IV infusion was started. The balloon was inflated repeatedly, gradually increasing the pressure (Figure 3). There was no significant tracheal expansion.

**Figure 4:**
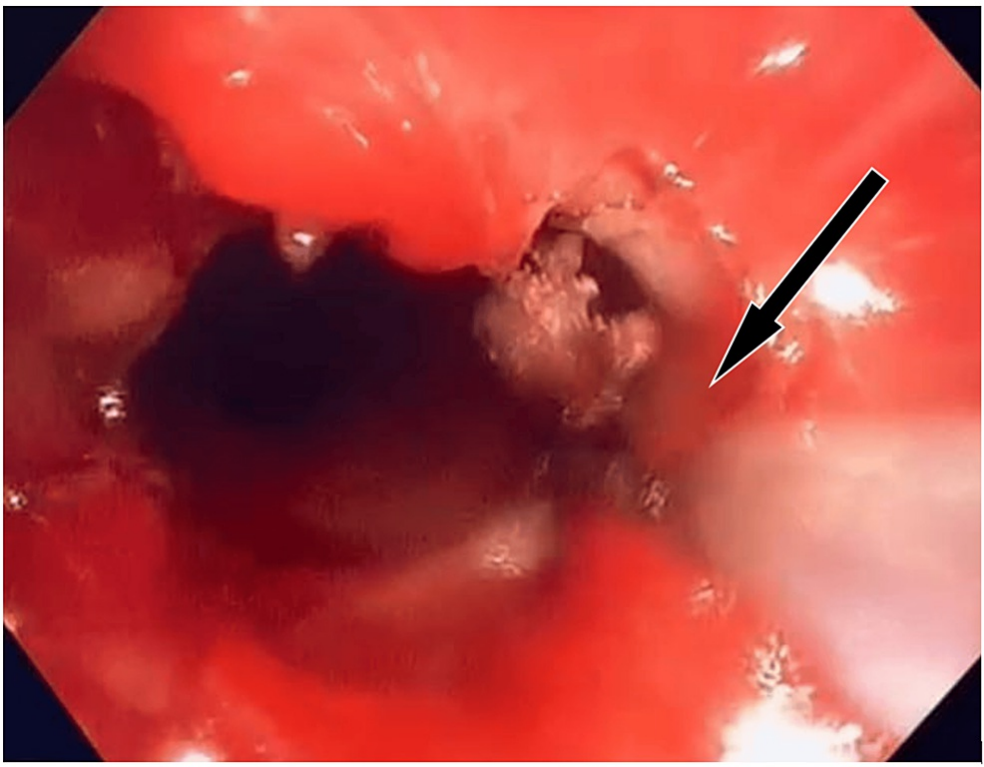
CO2 Laser was introduced through the bronchoscope. As the airway FiO_2_ dropped below 21%, endoscopic CO_2_ laser cauterization was carried out at the stenotic site to increase the tracheal diameter.

**Figure 5:**
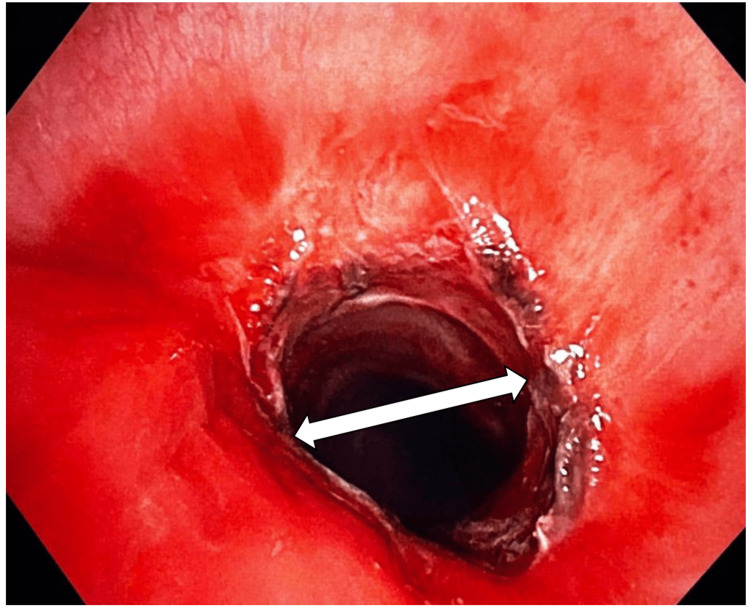
Subglottic area of the trachea post-procedure. After balloon dilatation and cauterization through the flexible bronchoscope, there is a significant dilatation of the tracheal diameter.

To minimize burns on the patient's outside body, normal laser safety precautions were carefully followed before employing the laser, such as covering the patient with damp towels and eye pads. Fresh gas flow was increased to 10 L per minute to speed up the clearance of oxygen in the airway, and FiO_2_ was dropped to 21% to reduce the risk of fire in the airway. These precautions have been proven to be sufficient in ensuring that the airway's FiO_2_ levels have fallen below the laser-use safety threshold (Figure 4). If the patient had reduced lung reserve, the procedure was paused and intermittent reoxygenation with 100% FiO_2_ was performed. After maximum cauterization of the stenotic site with laser, a balloon dilatation was performed. Notable improvement was observed in the tracheal diameter (Figure 5). Then 0.4 mg/mL of mitomycin C was applied at the dilatation site for 4 minutes, and the patient was reversed and the laryngeal airway was removed. On reversal, adequate spontaneous respiration was noted with no stridor and no subcostal and suprasternal retractions. Quiet and unobstructed respiration was established.

The patient was shifted to recovery on a Hudson mask with 6 L of oxygen flow maintaining 100% SpO_2_. The patient's symptoms of dyspnea and stridor improved markedly. Once the patient was comfortable and there was no respiratory distress, she was shifted to the ward on room air and eventually discharged.

## Discussion

Tracheal cuff stenosis mainly occurs due to loss of regional blood flow. The mucosa that is between the inflated ET tube cuff and the tracheal cartilage suffers ischemia if the cuff is inflated above mucosal capillary pressure (30 mm of Hg) of the trachea. Prolonged ischemia can cause damage to the tracheal cartilage and ulceration, and during the healing process, it may undergo fibrotic changes, leading to an increase in tracheal stenosis [14,15].

For a varied amount of time, these individuals may not exhibit any symptoms, but later they may experience dyspnea and difficulty in expectoration, which can worsen with effort and lead to airway obstruction and the formation of a stenosis. If the original diameter starts reducing to less than 30%, then symptoms start presenting [16].

The case discussed above is a recurrence of post-intubation tracheal stenosis, where there are chances of loss of structural integrity of tracheal cartilage resulting in a weak airway [17]. Under general anesthesia, this floppy airway can be unmasked, and the changes in transtracheal pressure gradient that are typically compensated in an awake individual are lost, which may result in the trachea collapsing completely. Owing to all these possibilities, the option of awake insertion of I-gel with tropical anesthesia and slight sedation was planned.

With the help of supraglottic devices such as I-gel, the laryngeal inlet is completely sealed off, creating a closed system that permits end-tidal CO_2_ (EtCO_2_) monitoring, and the patient can be maintained either on spontaneous or positive pressure ventilation. In contrast to ET intubation, the I-gel allows for good hemodynamic stability, lower anesthetic requirement, and less coughing and sore throat post-operatively. It makes it easier to optimize airway management for both maintaining intraoperative ventilation and comfortable surgical access for airway surgery [18].

Unlike apneic ventilation, where the patient's apneic duration limits the surgeon's operating time and necessitates work stoppage to ensure appropriate oxygenation, the I-gel technique allows the surgeon to use numerous modalities without interruption.

The I-gel is useful in patients who can be maintained on spontaneous ventilation, in patients with trismus or limitations in neck extension, obese patients, or patients with pulmonary comorbidities such as an increase in intra-pulmonary shunting. When jet ventilation is used in these patients, they have a risk of sudden drop in oxygen saturation. It also has a potential for tissue movement and decreased visualization due to intermittent vibration. The use of laser treatment with THRIVE is still under investigation [11].

I-gel-assisted endoscopic treatment of airway stenosis would be safe and efficacious as it has a low complication rate in comparison to published results of alternative methods for managing subglottic stenosis [19].

## Conclusions

Certainly, anesthesiologists must stay vigilant and maintain close communication with the interventional pulmonologist during airway procedures. Constant adjustments are needed to optimize patient oxygenation and ventilation, and anesthesiologists should be well-prepared with a backup plan tailored to the severity of any potential stenosis-related complications.

Awake insertion of a supraglottic airway device in our patient was beneficial, and the tracheal stenosis was successfully expanded. This would also be beneficial in areas where resources and specialty facilities are scarce. To establish this as a definitive method of airway management in cases of tracheal stenosis, further research and exploration are needed, and it is also necessary to know the degree and level of stenosis up to which this technique can be applied.
